# Secukinumab effectively manages ankylosing spondylitis in a hemodialysis patient without side effects 

**DOI:** 10.5414/CNCS111717

**Published:** 2025-09-04

**Authors:** Zewen Zhao, Yuhe Yin, Xiaoying Dong, Qingqing Gao, Haowen Lin, Siqi Peng, Yiming Tao, Sichun Wen, Bohou Li, Qiong Wu, Renwei Huang, Sijia Li, Ting Lin, Hao Dai, Zhuo Li, Lixia Xu, Jianchao Ma, Feng Wen, Zhonglin Feng, Shuangxin Liu, Yanhai Cui

**Affiliations:** 1 Department of Nephrology, Guangdong Provincial People’s Hospital, Guangdong Academy of Medical Sciences, Southern Medical University, Guangdong,; 2 Guangdong Cardiovascular Institute, Guangdong Provincial People’s Hospital, Guangdong Academy of Medical Sciences,; 3 Department of Radiology, Guangdong Provincial People’s Hospital, Guangdong Academy of Medical Sciences, Guangdong, China *Co-first authors: Zewen Zhao, Yuhe Yin, Xiaoying Dong

**Keywords:** ankylosing spondylitis, secukinumab, hemodialysis, reneal replacement, biologic therapy

## Abstract

Introduction: Ankylosing spondylitis (AS) is a chronic, progressive inflammatory disease that primarily affects the spine and sacroiliac joints. In recent years, biologic agents have gained increasing popularity in the treatment of AS due to their high targeting specificity and favorable side effect profiles. Among these, secukinumab has emerged as an effective treatment option. However, the safety and efficacy of secukinumab in patients undergoing hemodialysis has not yet been thoroughly verified. Case report: Here, we report the successful treatment of AS with secukinumab in a 36-year-old male patient undergoing hemodialysis. The patient presented with recurrent lumbosacral pain and tested positive for HLA-B27. After treatment, significant improvements were observed in both the imaging characteristics of the synovial joints and the patient’s symptoms. Conclusion: This case suggests that secukinumab may have a positive effect on AS in patients on hemodialysis, without apparent adverse effects.

## Introduction 

Ankylosing spondylitis (AS) is a prevalent chronic inflammatory disease classified under rheumatic immune disorders. The primary sites of pathology are the axial joints, with the sacroiliac joint being most commonly affected. Other involved sites include the spine, peripheral joints, and entheses. In severe cases, spinal deformity and ankylosis may develop. Approximately 90% of AS patients test positive for HLA-B27. A positive HLA-B27 result significantly increases the likelihood of an AS diagnosis [1]. Previous studies have demonstrated a close association between interleukin-17 (IL-17) and AS, with elevated IL-17 levels observed in AS patients [2]. AS can present with multisystem involvement, and the kidneys are frequently affected [3]. Renal involvement in AS patients has been attributed to various factors, such as the use of nephrotoxic medications, coexisting hypertension, and other complications [4]. Secukinumab is a fully human, high-affinity monoclonal antibody expressed in Chinese hamster ovary cell lines (CHO-HPT1). It specifically binds to interleukin-17A (IL-17A), neutralizing its cytokine activity. Secukinumab belongs to the IgG1/κ isotype subclass antibody [5]. As an IgG monoclonal antibody with a relatively large molecular size, secukinumab is predominantly metabolized in the reticuloendothelial system rather than being excreted via the kidneys. After being internalized through endocytosis or receptor-mediated endocytosis, it undergoes lysosomal degradation within cells, breaking down into amino acids [6, 7]. However, currently, there is limited information on the use of secukinumab for treating AS in patients on hemodialysis. This article aims to document the successful administration of secukinumab in a single AS patient undergoing hemodialysis, during which no adverse effects were observed. 

## Case report 

**Figure 1.**
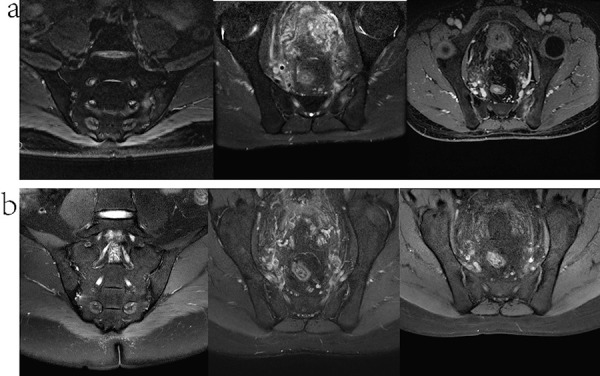
MR manifestations. a: Secukinumab treatment before the joint surface, bone marrow, synovial involvement. b: The involvement of articular surface, bone marrow, and synovium was alleviated after 1 year of secukinumab treatment.

**Figure 2.**
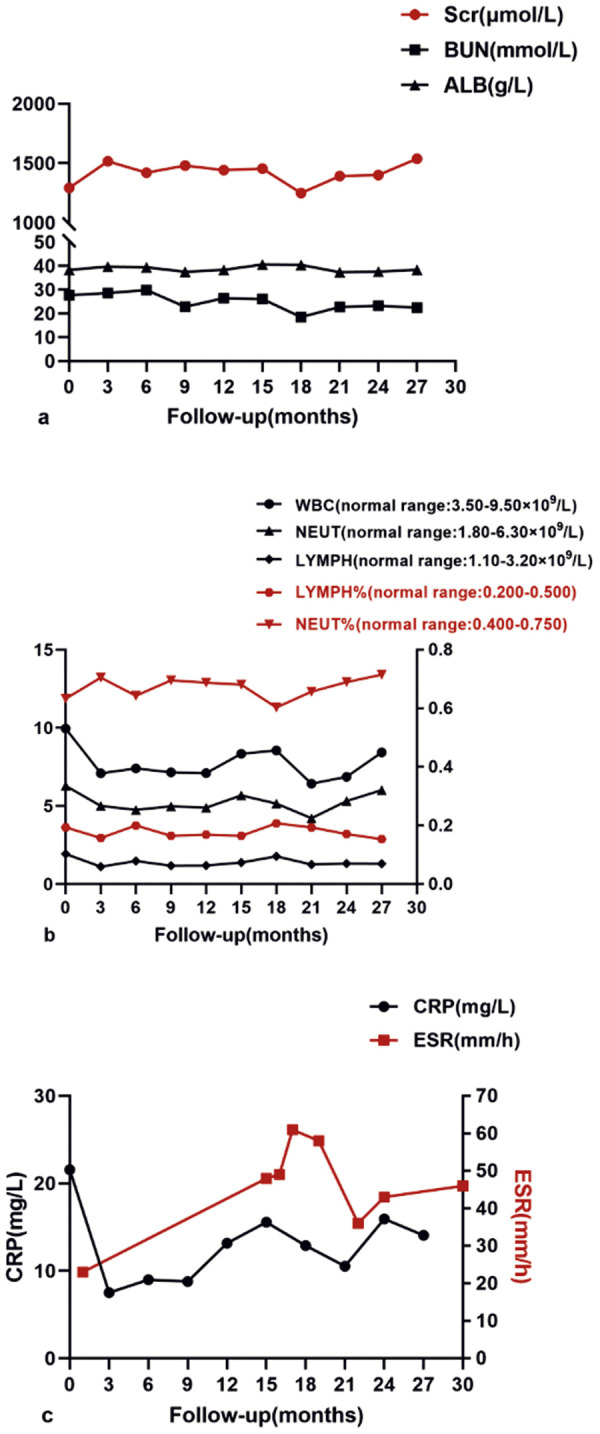
Laboratory examination. a: During treatment, serum creatinine, blood urea nitrogen and albumin levels were stable. b: Changes in white blood cell, lymphocyte, and neutrophil counts were observed during the therapy period. c: C-reactive protein decreased, and erythrocyte sedimentation rate tended to stabilize. ALB = Albumin; Scr = serum creatinine; BUN = blood urea nitrogen; WBC = white blood cell; NEUT = neutrophil; LYMPH = lymphocyte; CRP = C-reactive protein; ESR = erythrocyte sedimentation rate.

A 36-year-old male patient, who had been undergoing hemodialysis for 13 years due to primary glomerulonephritis, presented with recurrent lumbosacral pain and morning stiffness 2 years ago. These symptoms generally subsided with physical activity, although they disrupted his sleep quality to some extent. Notably, the HLA-B27 test yielded a positive result. C-reactive protein (CRP) level was measured at 21.56 mg/L, and the erythrocyte sedimentation rate (ESR) was 23 mm/h. Magnetic resonance imaging (MRI) scans revealed subtle, patchy abnormalities beneath the joint surfaces. These abnormalities exhibited mild hyperintensity on T1WI-FS and mixed high – low signals on T2WI. Post-enhancement scans indicated mild enhancement in these patches, suggesting bone marrow edema and inflammation, which was consistent with AS. Based on the patient’s comprehensive clinical profile, a diagnosis of AS was confirmed. He was initiated on weekly secukinumab therapy at a dose of 150 mg. After receiving 4 doses, the frequency was adjusted to once monthly, and this regimen was maintained for 2 years. After 1 year of treatment, coronal T2WI fat-suppression sequences showed the resolution of articular bone marrow edema. Additionally, enhanced fat-suppression sequences demonstrated the disappearance of both articular and synovial enhancement, indicating the resolution of inflammation (Figure 1). The patient experienced a significant improvement in pain symptoms. Throughout the treatment course, CRP levels exhibited a consistent downward trajectory. In contrast, the erythrocyte sedimentation rate (ESR) remained relatively stable, fluctuating within the range of 40 – 60 mm/h. Notably, during secukinumab therapy for AS in this hemodialysis patient, no adverse effects were observed. Moreover, his creatinine, blood urea nitrogen (BUN), and neutrophil percentage levels remained stable throughout the treatment period (Figure 2). 

## Discussion 

In this case, a hemodialysis patient with a confirmed diagnosis of AS underwent secukinumab therapy, which resulted in significant improvement in lumbosacral pain. MRI revealed a notable reduction in sacroiliac joint swelling and inflammation. No apparent side effects were reported during the treatment. Infection, environmental stimuli, genetic predisposition, and immune disruptions can initiate AS or contribute to its progression. Among these factors, the immune system plays a pivotal role in AS, involving diverse immune cells, cytokines, and antibodies. Spinal pathology in AS is characterized by altered bone formation and osteolysis, accompanied by immune alterations. Specifically, IL-17, secreted by CD4+ T cells, mast cells, and granulocytes, enhances receptor activator of nuclear factor-κ B (RANK) expression in osteoclast precursors, promoting osteoclast differentiation and bone loss. Additionally, IL-17 exacerbates local inflammation by inducing tumor necrosis factor-α (TNF-α) secretion [8, 9]. According to the 2009 Assessment of SpondyloArthritis international Society (ASAS) axial spondyloarthritis (SpA) classification criteria, a diagnosis of axial SpA can be made when a patient presents with back pain lasting for more than 3 months, combined with imaging evidence of sacroiliitis, along with any one of the SpA features such as HLA-B27 positivity, elevated CRP and ESR, inflammatory back pain, or psoriasis [10]. In this case, the 36-year-old man was definitively diagnosed with AS based on his history of recurrent lumbosacral pain, MRI findings indicating sacroiliitis, a positive HLA-B27 test result, and elevated levels of CRP and ESR. Traditional therapies for AS include nonsteroidal anti-inflammatory drugs (NSAIDs) and disease-modifying anti-rheumatic drugs (DMARDs). However, as our understanding of the pathogenesis of AS continues to deepen, the application of biological DMARDs in its treatment has ushered in a new era. Specifically, TNF inhibitors and IL inhibitors are increasingly being used [8]. Prolonged NSAID exposure is linked to an increased risk of cardiovascular events, gastrointestinal ulcers and their complications, nephrotoxic adverse effects, and deranged liver enzyme profiles [11]. Critically, in patients with end-stage renal disease (ESRD), NSAID use confers a markedly heightened risk of both cardiovascular events and major bleeding episodes [12]. The most prevalent adverse effects associated with TNF inhibitors are injection site reactions (ISRs), and they also increase the risk of opportunistic infections [13]. IL-17, a type of pro-inflammatory cytokine secreted by various immune cells, facilitates bone growth and regeneration by stimulating the proliferation and differentiation of osteoclasts. Secukinumab is a fully human monoclonal antibody that targets interleukin-17A. By disrupting the normal functional activity of IL-17A, it has the potential to mitigate inflammatory responses and impede the progression of bone destruction. Its side effects include an elevated risk of infections, dermatological reactions, ISRs, and inflammatory bowel disease (IBD) [14]. In a randomized, double-blind phase 3 clinical trial, secukinumab demonstrated rapid, substantial, and enduring improvement in the signs and symptoms of active AS. When administered as a 150-mg subcutaneous injection, secukinumab achieved a peak plasma concentration (t_max_) at 5.5 days and had a half-life of ~ 27 days. This pharmacokinetic profile supports a monthly dosing regimen to maintain an effective therapeutic concentration in the body. Moreover, a 150-mg dose of secukinumab was found to inhibit IL-17A in over 85% of patients, reaching a plateau effect in terms of its inhibitory action. Although both the 300-mg and 150-mg doses of secukinumab proved to be efficacious and well-tolerated, the use of higher doses did not confer additional clinical benefits. Consequently, the 150-mg dose can be considered as an optimal choice, which has the potential to reduce treatment costs [15]. However, there is a notable lack of understanding regarding the use of secukinumab, particularly in the context of renal impairment in AS patients. Several case studies have documented successful treatments of severe hidradenitis suppurativa and erythrodermic psoriasis with secukinumab in dialysis patients, without apparent adverse effects [16, 17, 18, 19]. Given these observations, secukinumab may emerge as a viable therapeutic alternative for AS patients undergoing dialysis. Nevertheless, the available data concerning the efficacy, safety, and long-term outcomes of secukinumab in patients with end-stage renal disease are limited, with no reported cases to date. Therefore, further research is imperative to elucidate the potential role of secukinumab in this patient population. 

We report a case of a hemodialysis patient with AS who was sensitive to new immunosuppressants and was treated with secukinumab. The patient’s sacroiliac joint pain was relieved, and there were no adverse reactions, especially no infections. The results suggest that secukinumab may have advantages in the treatment of hemodialysis combined with AS. However, the inherent limitations of this case must be recognized, including the need for long-term monitoring to assess the persistence of response, potential drug resistance to secukinumab, and the risk of disease progression. In addition, there are still doubts about the efficacy of secukinumab monotherapy compared to other immunosuppressive combination therapies, the full spectrum of its side effects, and the impact of serum biomarkers such as IL-17 on treatment outcomes. 

## Conclusion 

Secukinumab, a new anti-interleukin-17 biologic, effectively treats ankylosing spondylitis in a hemodialysis patient, with no apparent side effects in this case. It shows potential as a treatment option but requires further study. 

## Statement of ethics 

This retrospective review of patient data did not require ethical approval in accordance with local guidelines. This study protocol was reviewed and the need for approval was waived by Ethics Committee of Guangdong Provincial People’s Hospital. Written informed consent was obtained from the patient for publication of this case report and accompanying images. Written informed consent was obtained from the patient for publication of this case report and any accompanying images. 

## Data availability statement 

All data generated or analyzed during this study are included in this article. Further inquiries can be directed to the corresponding author. 

## Authors’ contributions 

Shuangxin Liu, Yanhai Cui, and Zhonglin Feng designed this study. Zewen Zhao, Yuhe Yin, and Xiaoying Dong were responsible for the data collection and analysis and wrote the manuscript. Qingqing Gao, Haowen Lin, Siqi Peng, Yiming Tao, Sichun Wen, Bohou Li, Qiong Wu, Renwei Huang, Sijia Li, Ting Lin, Hao Dai, Zhuo Li, Lixia Xu, and Jianchao Ma contributed to the follow-up study. Shuangxin Liu, Yanhai Cui, and Zhonglin Feng served as lead medical writers and illustrators for this manuscript. All authors reviewed the manuscript and signed off on its accuracy. 

## Funding 

This study was supported by the National Natural Science Foundation of China (No. 81870508, 81873616, and 82170730), Project supported by the KRT Plan of Guangdong Medical Development Foundation (No. 20240103, 20240105), and Natural Science Foundation of Guangdong Province (No. 2022A1515012374 and No. 2023A1515010024). 

## Conflict of interest 

The authors declare they have no conflict of interest.
